# Cordycepin Augments the Efficacy of Anti-PD1 against Colon Cancer

**DOI:** 10.3390/biomedicines12071568

**Published:** 2024-07-15

**Authors:** Wen-Kuei Chang, Yen-Ting Chen, Chin-Ping Lin, Chia-Jung Wang, Hui-Ru Shieh, Chih-Wen Chi, Tung-Hu Tsai, Yu-Jen Chen

**Affiliations:** 1 Institute of Traditional Medicine, School of Medicine, National Yang Ming Chiao Tung University, Taipei 112, Taiwan; ym40711002.y@nycu.edu.tw; 2 Division of Pulmonary and Critical Care Medicine, Department of Internal Medicine, MacKay Memorial Hospital, Taipei 104, Taiwan; 3 Department of Medicine, MacKay Medical College, New Taipei 252, Taiwan; 4 Department Medical Research, MacKay Memorial Hospital, New Taipei City 251, Taiwan; 5 Graduate Institute of Acupuncture Science, China Medical University, Taichung 404, Taiwan; 6 Department of Chemistry, National Sun Yat-Sen University, Kaohsiung 804, Taiwan; 7 School of Pharmacy, Kaohsiung Medical University, Kaohsiung 807, Taiwan; 8 Department of Medical Research, China Medical University Hospital, Taichung 404, Taiwan; 9 Department of Radiation Oncology, MacKay Memorial Hospital, Taipei 104, Taiwan

**Keywords:** cordycepin, adenosine, immune checkpoint inhibitor, CT26, anti-programmed cell death 1 (anti-PD1), tumor microenvironment

## Abstract

Colon cancer has a poor clinical response to anti-PD1 therapy. This study aimed to evaluate the effect of cordycepin on the efficacy of anti-PD1 treatment in colon cancer. The viability of CT26 mouse colon carcinoma cells, cell-cycle progression, morphology, and the expression of mRNA and protein were assessed. A syngeneic animal model was established by implanting CT26 cells into BALB/c mice for in vivo experiments. Multi-parameter flow cytometry was used to analyze the splenic cell lineages and tumor microenvironment (TME). The in vitro data revealed that cordycepin, but not adenosine, inhibited CT26 cell viability. The protein, but not mRNA, expression levels of A2AR and A2BR were suppressed by cordycepin but not by adenosine in CT26 cells. The combination of cordycepin, but not adenosine, with anti-PD1 exhibited a greater tumor-inhibitory effect than anti-PD1 alone as well as inhibited the expression of A2AR and A2BR in splenic macrophages. In the TME, the combination of cordycepin and anti-PD1 increased the number of CD3+ T cells and neutrophils and decreased the number of natural killer (NK) cells. Overall, cordycepin augmented the antitumor effects of anti-PD1 against mouse colon carcinoma cells and inhibited the expression of the adenosine receptors A2AR and A2BR in splenic macrophages and intratumoral NK cells.

## 1. Introduction

Immunotherapy is widely used as an antitumor treatment; however, its low response rate limits its clinical application, especially in colon cancer. Although programmed cell death-ligand 1 (PD-L1) and programmed cell death 1 (PD1) inhibitors have been approved for the treatment of advanced colorectal cancer (CRC) with a deficiency in mismatch repair (dMMR) and/or high microsatellite instability (MSI), patients with proficient mismatch repair (pMMR) and microsatellite stable (pMMR/MSS) CRC, accounting for more than 80% of all CRC cases, exhibit poor responses to immune checkpoint inhibitors [1,2]. Combination therapy with immune checkpoint inhibitors and chemotherapy has shown promising efficacy [3]; however, the toxicities after such treatments are greater than those of either immunotherapy or chemotherapy alone. Therefore, there is an unmet need for a new combination therapy with immune checkpoint inhibitors and medicines other than those used for chemotherapy that augments the efficacy of anti-PD1 and enhances the antitumor response with tolerable side effects [4,5,6], thereby improving the survival and quality of life of patients.

Cordycepin, the bioactive constituent of *Cordyceps sinensis*, acts as an adenosine analog and exerts anti-oxidative [7,8], anti-inflammatory [9,10], immunomodulatory [11], and anti-cancer effects [12,13,14,15,16]. The inhibitory effects of cordycepin on colon cancer via various mechanisms have been well documented [17,18,19], including the inhibition of colon cancer cell growth after binding to the adenosine A3 receptor (A3R) [20]. Cordycepin induces cancer cell apoptosis via the adenosine pathway through the adenosine 2A receptor (A2AR) [21] or A3R [22]. The binding of adenosine 1 receptor (A1R) and adenosine 2B receptor (A2BR) to their agonists is associated with the impairment of glioblastoma growth [23], induction of programmed cell death ligand 1 (PD-L1) expression, and decreased infiltration of natural killer (NK) cells [24]. Although the blockade of adenosine receptors increases the efficacy of anti-PD-1 and modulates the immune response [25,26,27,28,29], only a few studies have investigated the effects of cordycepin on immune checkpoint inhibitors, as well as the efficacy of combined anti-cancer treatment with cordycepin and immune checkpoint inhibitors.

We hypothesized that cordycepin, an adenosine derivative, modulates the efficacy of immunotherapy via adenosine signaling in immune cells. To verify this, we established a syngeneic tumor implantation animal model and examined the effects of cordycepin on the adenosine signaling pathway in mouse CT26 tumor cells, a colorectal carcinoma cell line derived from BALB/c mice expressing PD-1. We aimed to explore antitumor immunity and the effect of cordycepin on the efficacy of anti-PD-1 immunotherapy. We also evaluated the tumor microenvironment (TME) after the combined treatment with cordycepin and anti-PD1.

## 2. Materials and Methods

### 2.1. Cell Culture

CT26 mouse colon carcinoma cells were purchased from the American Type Culture Collection (Manassas, VA, USA) and cultured in Roswell Park Memorial Institute-1640 medium supplemented with L-glutamine, 25 mM HEPES (Merck, Darmstadt, Germany), 10% fetal bovine serum (Hyclone, Logan, UT, USA), and 1% penicillin at 37 °C in a 5% CO_2_ incubator. Cells were maintained in the exponential growth phase. 

### 2.2. Cell Viability Analysis

A trypan blue exclusion assay was used to assess cell viability. CT26 cells were treated with various concentrations of adenosine (0, 50, and 100 μM) obtained from Tokyo Chemical Industry Co., Ltd. (TCI) (Tokyo, Japan), and cordycepin (0, 25, and 50 μM; TCI, Tokyo, Japan) for 24, 48, and 72 h. Then, the number of viable cells was determined using a trypan blue exclusion assay.

### 2.3. Cell Cycle Analysis

CT26 cells (10^5^ cells/mL) were seeded in 3.5 cm dishes for 24 h, and then treated with adenosine (0, 50, and 100 μM) in phosphate-buffered saline (PBS) (AMRESCO, Solon, OH, USA) and cordycepin (0, 25, and 50 μM) in PBS for 24 h. Following the administration of medication, the CT26 cells were separated and subjected to washing with PBS and fixation with 70% ethanol. After the above procedures, followed by washing and re-suspension in cold PBS, the cells were incubated with 10 mg/mL RNase (Sigma-Aldrich, Saint Louis, MO, USA) and 1 mg/mL propidium iodide (PI) (Sigma-Aldrich, Saint Louis, MO, USA) at 37 °C in the absence of light for 30 min. A flow cytometer (BD FACSCalibur; Becton Dickinson, Lincoln Park, NJ, USA) was used to analyze the samples. Finally, the CellQuest Pro version 6.1 software (Becton Dickinson, Lincoln Park, NJ, USA) was used to evaluate the percentage of CT26 cells in the G0/G1, S, and G2/M phases. All experiments were performed in triplicates.

### 2.4. Morphological Observation by Light Microscopy

CT26 cells were cultured in 6-well plates followed by incubation for 24 and 48 h. After two PBS washes, the CT26 cells were stained using Liu’s stain solution A (Muto Pure Chemicals Co., Ltd., Bunkyou-Ku, Tokyo, Japan) for 45 s, followed by solution B for 90 s. Finally, cell morphological observations were performed using a BX51 light microscope (Olympus, Tokyo, Japan).

### 2.5. Western Blotting Analysis

After treatment with adenosine and cordycepin, whole-cell lysates were generated using a lysis buffer (Cell Signaling Technology, Danvers, MA, USA). A bicinchoninic acid protein assay kit (Bio-Rad Laboratories, Hercules, CA, USA) was used to quantify the proteins. Equal quantities of protein were electrophoresed on a 10% sodium dodecyl sulfate-polyacrylamide gel and transferred onto a polyvinylidene difluoride membrane. The membrane was blocked with 5% nonfat milk and immunoblotted with primary antibodies against A2AR (GeneTex, Irvine, CA, USA), A2BR (Abcam, Cambridge, UK), and α-tubulin (GeneTex, Irvine, CA, USA) at 4 °C overnight. After incubation with secondary antibodies (Jackson ImmunoResearch, West Grove, PA, USA), the membranes were developed using an enhanced chemiluminescence system (GeneTex). The results were examined using the Multigel-21 multifunctional gel imaging system (Top Bio Co., Lin Kou, New Taipei, Taiwan). As an internal control, α-tubulin expression was employed. The intensity of the immunoblot images was measured using the ImageJ 1.54 g (Wayne Rasband and contributors, NIH, Bethesda, MD, USA).

### 2.6. mRNA Expression Assessment

RNAzol RT reagent (Molecular Research Center, Inc., Cincinnati, OH, USA) was used to extract total RNA from CT26 cells. The RevertAid First Strand cDNA Synthesis Kit (Thermo Fisher Scientific, Waltham, MA, USA) was then employed to reverse-transcribe RNA (1 μg) cDNA. RT-qPCR was performed using the LightCycler 96 Real-Time PCR System (Roche). PCR cycles were carried out at 65 °C for 5 min, 42 °C for 60 min, 70 °C for 5 min, 1 cycle at 95 °C for 60 s, and 40 cycles at 95 °C for 15 s and then at 60 °C for 30 s. The following primers were used: A1 forward 5′-AGAACCACCTCCACCCTTCT-3′ and reverse 5′-TACTCTGGGTGGTGGTCACA-3’, A2A forward 5′-TCAACAGCAACCTGCAGAAC-3′ and reverse 5′-GGCTGAAGATGGAACTCTGC-3, ’A2B forward 5′-GCGAATAAAAGCTGCTGTCC-3′ and reverse 5′-CCTGGAGTGGTCCATCAGTT-3’, A3 forward 5′-TCTTGGCATTCCTTCTGCTT-3′ and reverse 5′-AGCCAGGGCTACACAGAGAA-3’, and actin forward 5′-GCCAACCGTGAAAAGATGAC-3′ and reverse 5′-GAGGCATA CAGGGACAGCAC-3’. Gene expression was normalized to that of actin in the same sample.

### 2.7. Syngeneic Tumor Implantation Model

All animal experiments were approved by the Experimental Animal Committee of MacKay Memorial Hospital in Taiwan (approval number: MMH-A-S-109-42) and carried out in compliance with the regulations. A 12 h light/dark cycle was implemented in a pathogen-free facility to house 30 male BALB/c mice at 5 weeks of age that were obtained from the National Laboratory Animal Center of Taiwan (Taipei, Taiwan). The subcutaneous inoculation of the mice’s right hindlimb was then carried out using CT26 cells (4 × 10^6^) in 50 μL PBS. Mice were randomly assigned to six groups after the tumors had grown for seven days to a mean diameter of roughly 5 mm: (1) control, (2) adenosine (50 mg/kg thrice a week via oral gavage), (3) cordycepin (50 mg/kg thrice a week via oral gavage), (4) intraperitoneal anti-mouse PD-1 (Bio X cell, Lebanon, NH, USA) injection (250 mcg per intraperitoneal injection every other day three times in total; RMP1-14, Bio X cell, Lebanon, NH, USA), (5) adenosine with anti-mouse PD-1, and (6) cordycepin with anti-mouse PD-1.

### 2.8. Evaluation of the Tumor Volume and Toxicity

The tumor volume and body weight of each mouse were recorded on alternate days by a single assistant. Tumor volume was recorded using the formula 1/2ab^2^, where a represents the largest diameter, and b represents the smallest diameter of the tumor, recorded using electronic calipers. Blood samples were obtained from the retro-orbital fossa, and the white blood cell count was measured using an automatic Coulter counter (HEMAVET HV950; Drew Scientific, Inc., Miami Lakes, FL, USA). Plasma alanine aminotransferase (ALT) and creatinine levels were measured using the Fuji Dri Chem Slide (FUJIFILM Corporation Asaka Technology Development Center, Minamiashigara-shi, Kanagawa, Japan) and detected using the Fujifilm DryChem NX-500 analyzer (FUJIFILM Corporation, Tokyo, Japan).

### 2.9. Flow Cytometry Analysis of Immune Cells

After treatment, mice were euthanized with xylazine (10 mg/kg) and ketamine (100 mg/kg). The tumor and spleen specimens were excised, cut into 2–4 mm pieces, and digested with a solution containing Liberase TM (25 μg/mL; Roche) and DNase I (1 0 μg/mL) at 37 °C for 30 min. Cells were sieved through a 70 μm cell strainer to dissociate and collect the single-cell suspensions. Red blood cells were lysed using ammonium chloride-potassium solution (Invitrogen, Waltham, MA, USA). The collected cells were suspended in an Fc receptor block (1 μg/1 × 10^6^ cells; BD Bioscience, San Diego, CA, USA) at 37 °C for 1 h to minimize nonspecific binding before staining with cell surface markers. To obtain single cells, small pieces of specimens were filtered with a 70 μm cell strainer and then using ACK lysis buffer to lyse red blood cell. The cells were then blocked using Fc BlockTM reagent (BD Bioscience, San Jose, CA, USA) to minimize non-specific binding for 1 h at 37 °C. The collected cells were stained with antibodies conjugated with the following fluorochromes for 20 min on ice: A2AR (FITC) (Santa Cruz, Dallas, TX, USA), A2BR (PE) (Bioss, Woburn, MA, USA), NKG2D (PE/Dazzle 594), Ly6G (PE/Cyanine7), CD8 (APC), F4/80 (APC/Cyanine7), (Brilliant Violet 421), CD45 (Brilliant Violet 510), CD11b (Brilliant Violet 605), CD4 (Brilliant Violet 650), and CD3 (Brilliant Violet 785) (BioLegend, San Diego, CA, USA). After washing, the cells were analyzed using a CytoFLEX 13-color cytometer (Beckman Coulter, Brea, CA, USA) and quantified using the CytExpert analysis software version 2.3.0.84 (Beckman Coulter). Immune profiles were defined as follows: CD3+ T cells (CD45+/CD11b−/CD3+), CD8+ T cells (CD45+/CD11b−/CD3+/CD8+), CD8+-A2AR(CD45+/CD11b−/CD3+/CD8+/A2AR+), CD8+-A2BR(CD45+/CD11b−/CD3+/CD8+/A2BR+), CD4+ T cells (CD45+/CD11b−/CD3+/CD4+), CD4+-A2AR(CD45+/CD11b−/CD3+/CD4+/A2AR+), CD4+-A2BR(CD45+/CD11b−/CD3+/CD4+/A2BR+), NKG2D+ T cells (CD45+/CD11b−/CD3+/NKG2D+), natural killer (NK) cells (CD45+/CD3−/Ly6G−/MHCII−/NKG2D+), NK-A2AR (CD45+/CD3−/Ly6G−/MHCII−/NKG2D+/A2AR+), NK-A2BR (CD45+/CD3−/Ly6G−/MHCII−/NKG2D+/A2BR+), neutrophils (CD45+/CD11b−/CD3−/Ly6G+), macrophage (CD45+/CD3−/Ly6G−/CD11b+/F4/80+), macrophage-A2AR (CD45+/CD3−/Ly6G−/CD11b+/F4/80+/A2AR+), and macrophage-A2BR (CD45+/CD3−/Ly6G−/CD11b+/F4/80+/A2BR+).

### 2.10. Statistical Analyses

Data are expressed as the mean ± standard deviation. Statistical comparisons were performed using one-way analysis of variance (ANOVA), followed by the least significant difference test for post hoc analysis. The association between the experimental group and control group regarding repeated measurements in this research was analyzed using repeated-measures ANOVA and a generalized estimated equation. All statistical analyses were conducted using IBM SPSS Statistics version 29.0.1.1 (IBM Corp., Armonk, NY, USA), SigmaPlot version 29.0, (Systat Software, Inc., San Jose, CA, USA), Prism version 8.3.0 (GraphPad Software, LLC, Boston, MA, USA), and ImageJ 1.54g (Wayne Rasband and contributors, NIH, USA). Significant differences between groups were indicated by *p* < 0.05.

## 3. Results

### 3.1. Cordycepin and Adenosine on Mouse Colorectal Carcinoma Cell Growth

**Figure 1:**
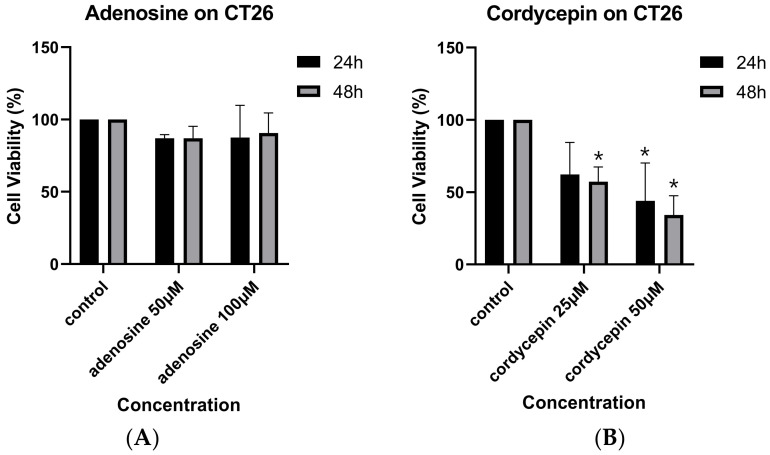
Cell viability of mouse colorectal carcinoma cells after administration with adenosine and cordycepin. The CT26 mouse colorectal adenocarcinoma cells were treated with 50 and 100 μM adenosine (**A**) and 25 and 50 μM cordycepin (**B**), respectively, for 24 and 48 h. Cell viability was measured via trypan blue assay. Data from two separate experiments are expressed as mean ± standard deviation (SD). Significant differences between the control cells and cells administered with adenosine and cordycepin are marked by * *p* < 0.05.

The effects of cordycepin and adenosine on the growth of mouse colorectal carcinoma cells are shown in Figure 1A and Figure 1B, respectively. Cordycepin, but not adenosine, reduced CT26 cell viability in a dose- and time-dependent manner. The half-maximal inhibitory concentration (IC50) of cordycepin was 39.95 μM at 24 h (Figure 1B), but the IC50 of adenosine was not reached at 24 or 48 h (Figure 1A).

### 3.2. Cordycepin and Adenosine on Cell Cycle of Mouse Colonrectal Carcinoma Cells

**Figure 2:**
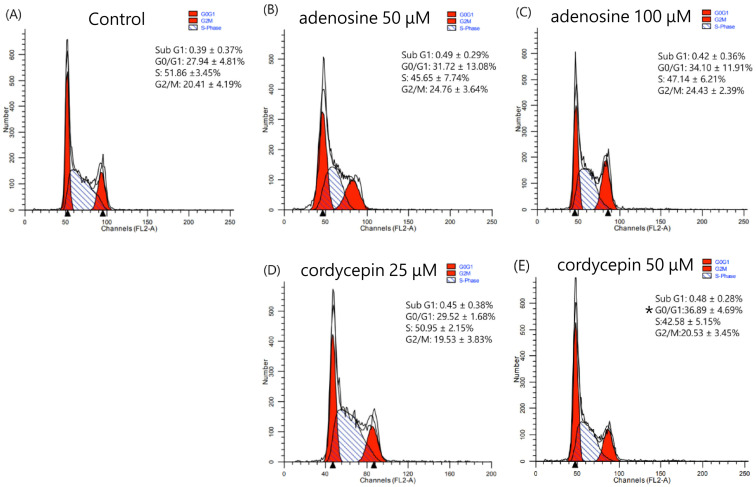
Cell cycle analysis of CT26 mouse colon carcinoma cells treated with 50 μM adenosine (**B**), 100 μM adenosine (**C**), 25 μM cordycepin (**D**), and 50 μM cordycepin (**E**) for 24 h. After treatment, the cells were washed with PBS, and fixed with 70% ethanol, and stained with propidium iodide. Cell cycle distribution was assessed via flow cytometry. Representative DNA histograms of colorectal cells treated with adenosine and cordycepin comparing to control group (**A**) are shown, and the expression percentage in each cell cycle phase is indicated in the panel. Data from four separate experiments are expressed as the mean ± SD. Significant differences between the control cells and cells administered with adenosine and cordycepin are marked by * *p* < 0.05. PBS: phosphate-buffered saline; SD: standard deviation.

The effects of cordycepin and adenosine on the cell cycle of colon carcinoma cells, CT26, are shown in Figure 2A–E. A DNA histogram analysis revealed that adenosine and cordycepin did not affect cell cycle distribution in CT26 cells, except that cordycepin, at 50 μM, increased the G0/G1 percentage (*p* = 0.034).

### 3.3. Cordycepin and Adenosine on Morphology of Colorectal Carcinoma Cells

**Figure 3:**
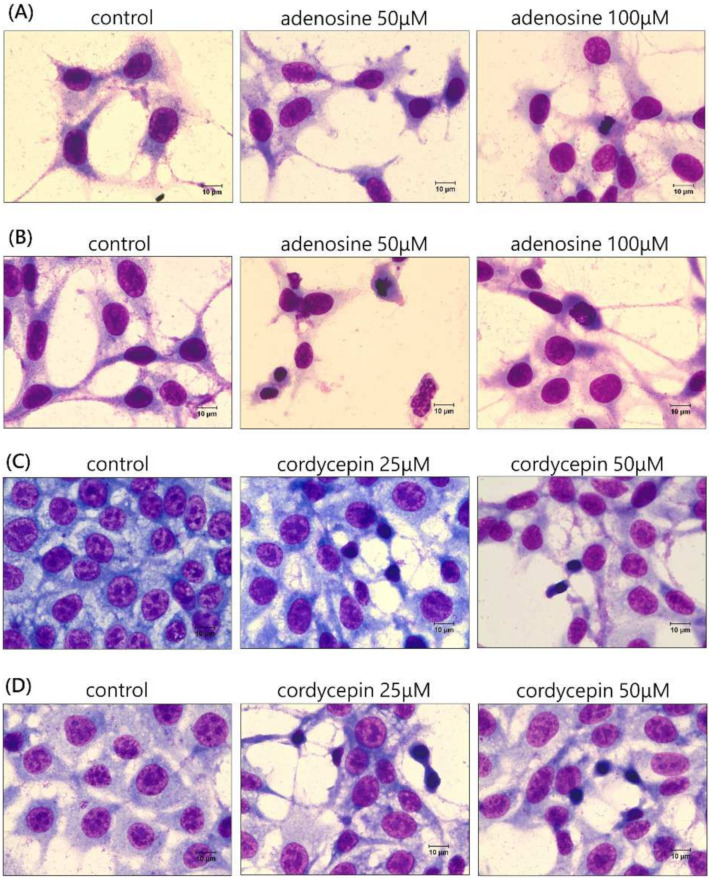
Morphology of CT26 colorectal carcinoma cells treated with 50 and 100 μM adenosine and 25 and 50 μM cordycepin for 24 h (**A**,**C**) and 48 h (**B**,**D**).

A morphology assessment of the CT26 mouse colorectal carcinoma cells via Liu’s staining revealed no significant changes in cell morphology after 50 and 100 μM adenosine and 25 and 50 μM cordycepin treatments for 24 and 48 h (Figure 3). Additionally, no significant apoptosis, vesicle formation, or swelling was observed in any of the groups.

### 3.4. Cordycepin and Adenosine on Adenosine Receptor mRNA Expression in Mouse Colorectal Carcinoma Cells

**Figure 4:**
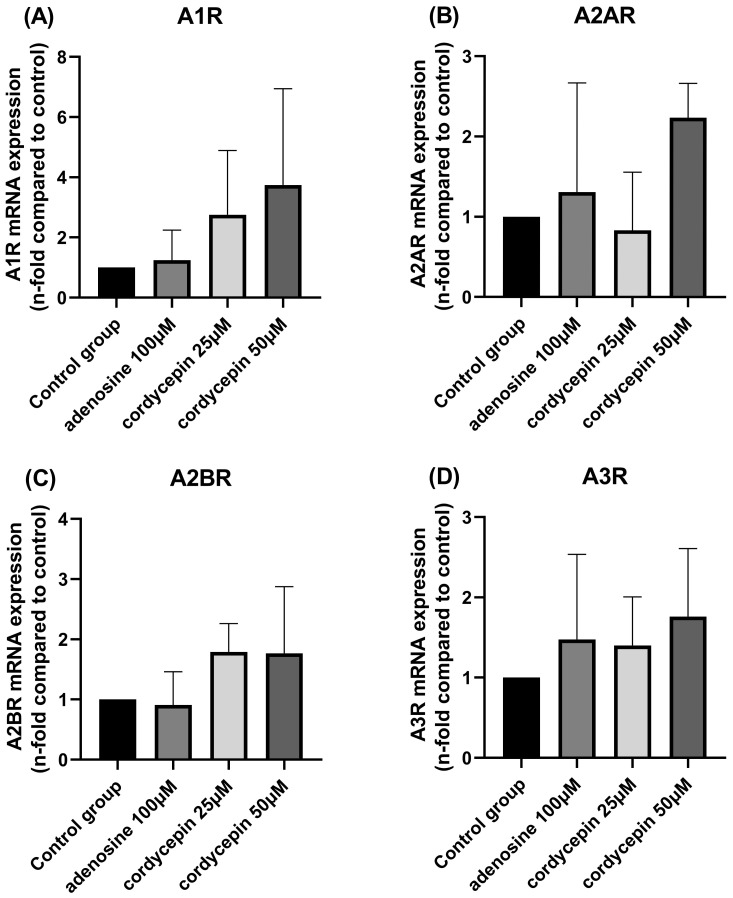
mRNA expression profiles in CT26 colorectal carcinoma cells after adenosine and cordycepin treatment. CT26 cells were grown in a monolayer and treated with 100 μM adenosine and 25 and 50 μM cordycepin for 24 h. The mRNA expression of A1R (**A**), A2AR (**B**), A2BR (**C**), and A3R (**D**) are shown. The results from each group are expressed as the mean ± SD.

The adenosine receptor mRNA expression levels in cells after treatment with adenosine and cordycepin are shown in Figure 4A–D. Compared with the control group, no significant differences were observed in the mRNA expression levels of adenosine receptors after adenosine and cordycepin treatment in CT26 colorectal carcinoma cells. 

### 3.5. Cordycepin Inhibited Molecular Target Protein Expression of A2AR and A2BR in Mouse Colorectal Carcinoma Cells

**Figure 5:**
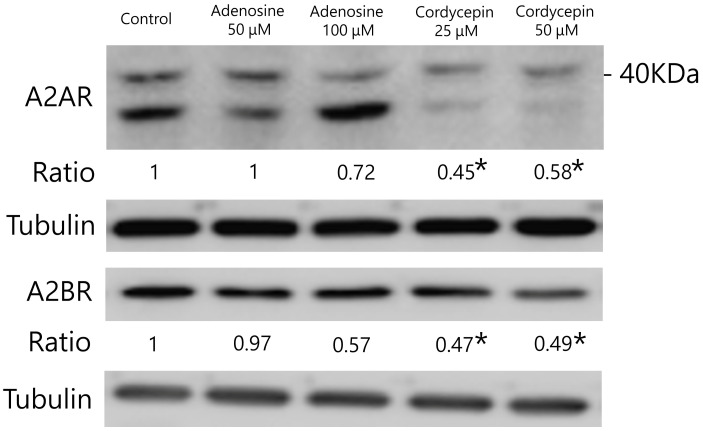
Effects of adenosine and cordycepin on the expression levels of putative target proteins in CT26 colorectal carcinoma cells. Cells were treated with 50 and 100 μM adenosine and 25 and 50 μM cordycepin for 24 h, lysed, and harvested. Equal amounts of proteins were subjected to immunoblotting to detect the A2AR and A2BR proteins. Representative blots of A2AR and A2BR proteins are shown. Densitometric analysis results of each band comparing to control group are shown as ratio. Significant differences between the control cells and cells treated with adenosine and cordycepin are indicated by * *p* < 0.05.

The protein expression levels of A2AR and A2BR in colorectal carcinoma cells are shown in Figure 5. In CT26 cells, the A2AR expression levels were significantly reduced after cordycepin (25 and 50 μM) treatment compared to those in the control group (*p* = 0.001 and 0.04, respectively). Similarly, the A2BR expression levels were reduced after cordycepin (25 and 50 μM) treatment compared to those in the control group (*p* = 0.024 and 0.031, respectively). However, no significant differences were observed in the A2AR and A2BR levels between the adenosine-treated and control groups.

### 3.6. Combination of Cordycepin and Anti-PD1 against Syngeneic CT26 Implanted Tumor In Vivo

**Figure 6:**
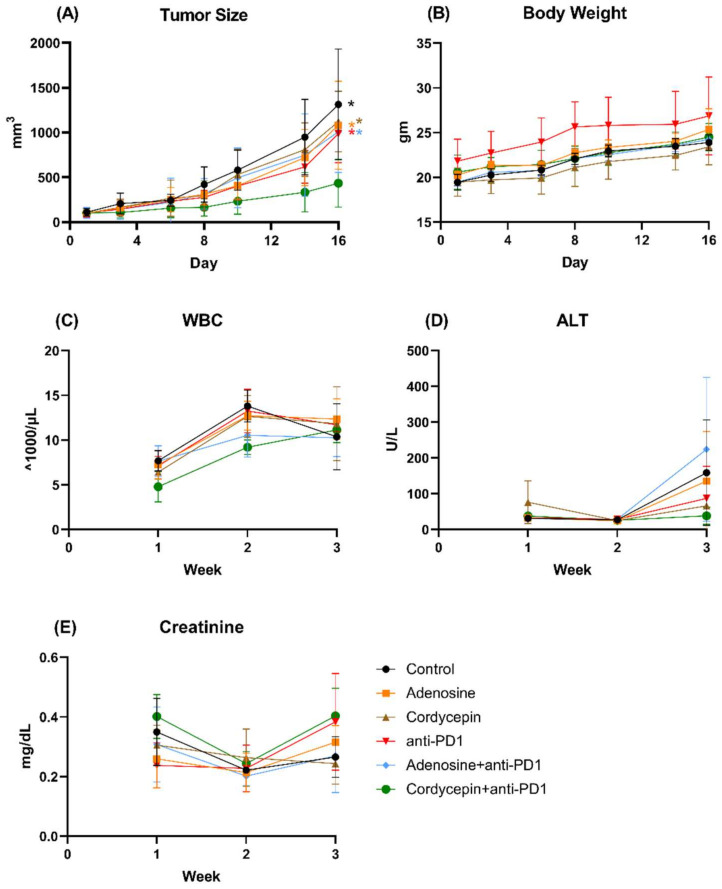
Therapeutic effects and toxicities of adenosine, cordycepin, anti-PD1, combination of adenosine and anti-PD1, combination of cordycepin and anti-PD1 in the CT26 syngeneic animal model. Tumoral growth in each group was evaluated via tumor size in mm^3^ (**A**). In vivo toxicities were detected via white blood cell counts (**B**), body weights (**C**), alanine transaminase levels representing liver function (**D**), and creatinine levels representing kidney function (**E**). The results from each group are expressed as the mean ± SD. The association between the control group and each experimental group showing statistical significance is expressed as * *p* < 0.05.

The combined effects of cordycepin and anti-PD-1 against syngeneic CT26 implanted tumors in vivo are shown in Figure 6. The in vivo experiments revealed that combination treatment with cordycepin and anti-PD-1 significantly reduced tumor size (Figure 6A) compared to combination treatment with adenosine and anti-PD-1, adenosine alone, cordycepin alone, and anti-PD-1 alone (*p* = 0.002, 0.002, 0.007, and 0.035, respectively). Between-group analyses of the body weight, WBC count, ALT levels, and creatinine levels revealed no significant differences (Figure 6B–E).

### 3.7. Analysis of Immune Cell Profiles in Tumor Microenvironment and Spleen In Vivo

**Figure 7:**
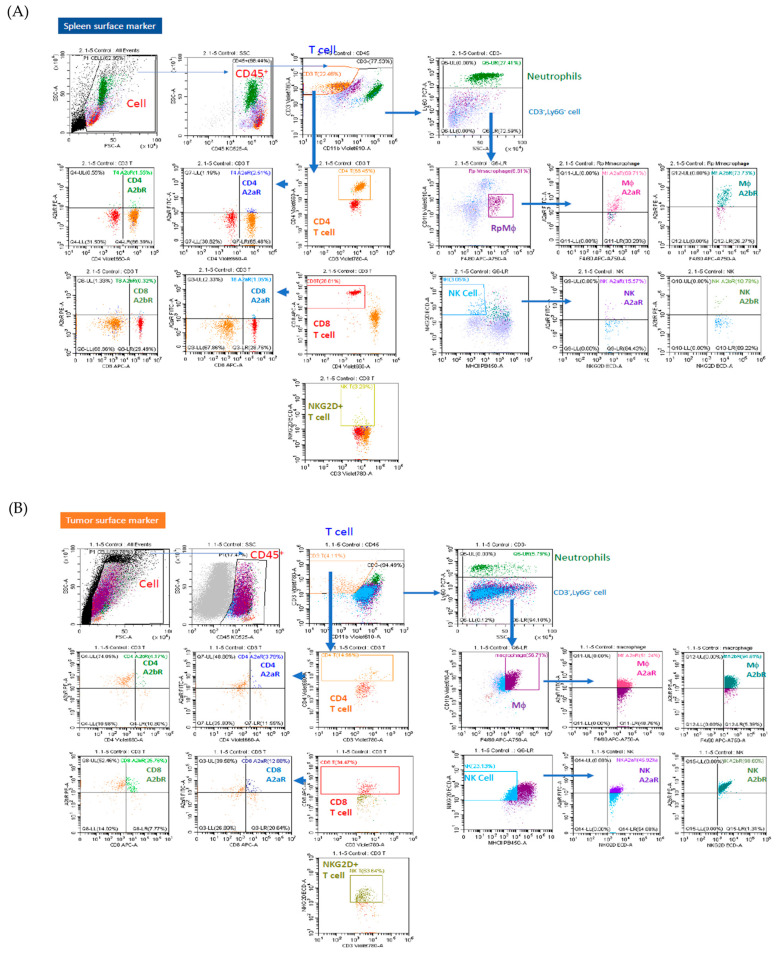

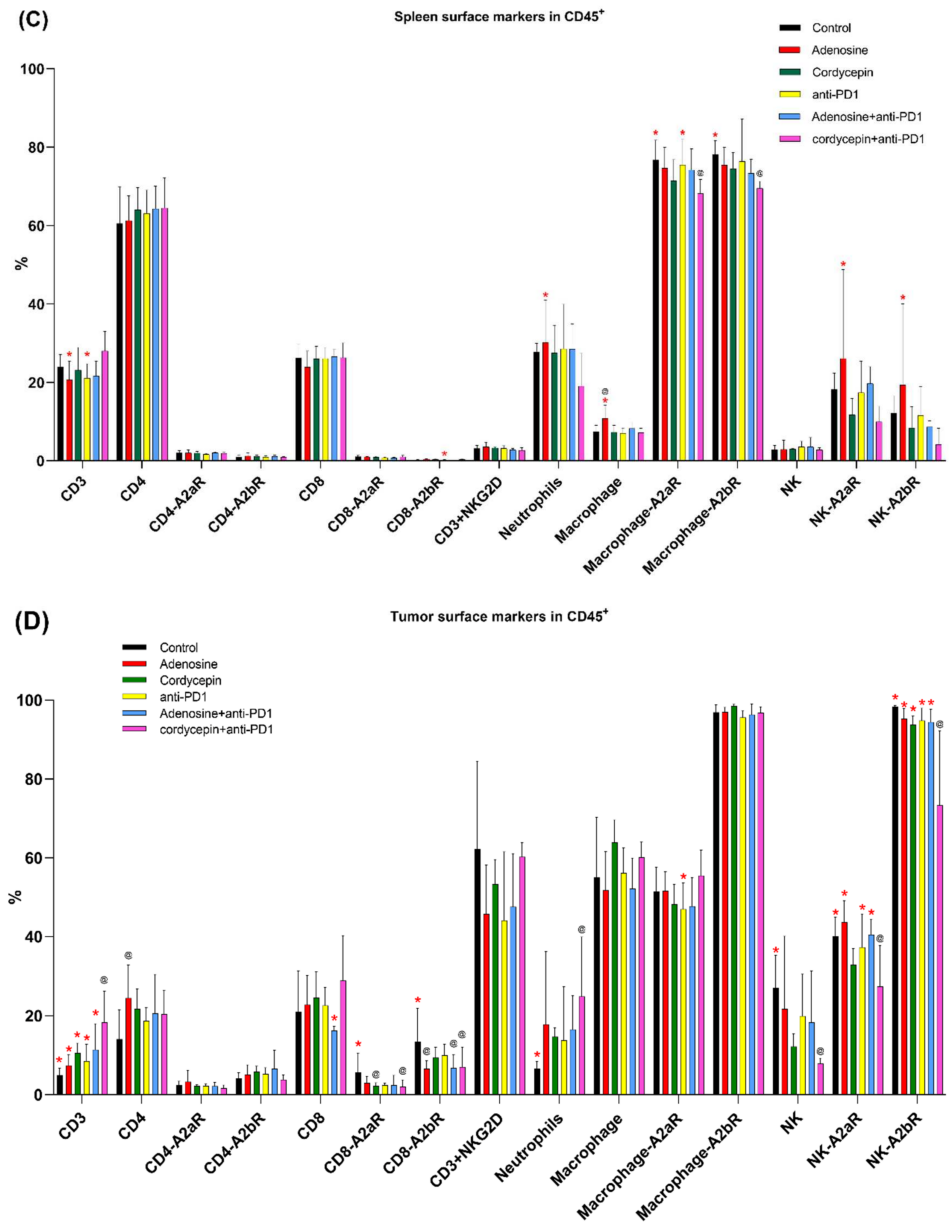
Expression profiles of circulating immune cells in the CT26 syngeneic animal model after treatment with adenosine, cordycepin, anti-PD1, the combination of adenosine and anti-PD1, and the combination of cordycepin and anti-PD1. Representative flow cytometric plots of each immune cell are shown and demonstrate the gating process of flow cytometry in spleen (**A**) and tumor (**B**) specimens. Analysis of different immune cells in spleen (**C**) and subcutaneous tumor (**D**) specimens in percentage on day 31 after the subcutaneous seeding of tumor cells in syngeneic mice model. The results from each group are expressed as the mean ± SD. The association between the control group and each experimental group showing statistical significance is expressed as @ *p* < 0.05. The association between the cordycepin plus anti-PD1 treatment group and any other group showing statistical significance is expressed as * *p* < 0.05.

Immune cell profiles of the TME and spleen in vivo are shown in Figure 7. The gating strategies for the flow cytometry analysis of the tumor and spleen are shown in Figure 7A and B, respectively. In the spleen, representing the mixed circulating cell and splenic immune cell lineages, the combination of cordycepin and anti-PD1 decreased the number of macrophages and inhibited the expression of A2AR and A2BR in macrophages compared to that in the controls (Figure 7C). In the TME, the combination of cordycepin and anti-PD1 increased the number of CD3+ T cells and neutrophils but decreased the number of NK cells compared to that in the controls. This combination treatment inhibited the expression of both A2AR and A2BR in NK cells (Figure 7D). In contrast, the combination of adenosine and anti-PD1 had no significant effect on these groups.

## 4. Discussion

In this study, we revealed a novel role of cordycepin in improving the immune checkpoint inhibitor response in mouse colon carcinoma cells. After the combination treatment with cordycepin and anti-PD1, the suppressive effect on CT26 cells was significantly higher than that of cordycepin alone, anti-PD1 alone, or the combination of adenosine and anti-PD1. Here, combination treatment with cordycepin and anti-PD1 did not cause significant changes in body weight, liver function, renal function, or WBC count in mice, indicating its therapeutic potential and safety.

The in vitro data showed that cordycepin inhibited cell viability with moderate bioactivity. Similar results have been reported by Deng et al. [30], who found that cordycepin inhibited the growth and migration of CT26 cells in a dose-dependent manner. Morphologically, neither cordycepin- nor adenosine-treated CT26 cells exhibited apoptosis, autophagy, necrosis, or mitosis. This suggests that cordycepin may inhibit cell viability, accompanied by cell death in an atypical manner. However, Deng et al. observed the promotion of apoptosis in CT26 cells after cordycepin administration [30]. Further investigations are warranted to clarify the detailed mechanism.

The Western blot analysis revealed that cordycepin significantly inhibited the expression of A2AR and A2BR in CT26 cells. Since the expression of these two receptors has been demonstrated to be negatively correlated with immune cell activation, whether this effect correlates with the development of immunogenic cell death in cancer cells remains to be elucidated. 

In the syngeneic colon cancer model, immune cell lineages in the circulation and TME were evaluated simultaneously. The most consistent and significant results were obtained with the combined cordycepin and anti-PD1 groups. Through combining anti-PD1 with cordycepin, but not adenosine, the following immune parameters were significantly altered: A2BR expression decreased in splenic macrophages; A2AR and A2BR expression levels decreased in intratumoral NK cells; and the numbers of intratumoral neutrophils and CD3+ lymphocytes increased. In the TME, NK cell numbers decreased after the combination treatment with cordycepin and anti-PD1; however, monotherapy with cordycepin or anti-PD1 alone did not exhibit such effects. A similar finding was reported by Wang et al., where high A2BR expression in oral squamous cell carcinoma was associated with a low infiltration of NK cells [24]. Suppressive effects on the generation of CD39+ NK cells via the inhibition of IL-15 signaling after A2A adenosine receptor ligation [31] and NK cell maturation have been well documented [26]. In our study, the downregulatory effect was not only observed in NK A2BR but also in NK A2AR expression after combination treatment with cordycepin and anti-PD1. Notably, this effect was not observed with cordycepin or adenosine treatment alone. Although the signals of NK cells were lower after combined cordycepin and anti-PD1 therapy in the TME, indicating lower infiltration of NK cells into tumors after such treatment, suppressed signals of A2AR and A2BR, both of which play inhibitory roles in NK cells, were observed after combination treatment with cordycepin and anti-PD1. Since a higher expression of A2AR and A2BR has been associated with tumor cell growth signals, the inhibition of NK cell cytotoxicity [23,31], and limiting the maturation of NK cells [26] in previous studies, the suppression of tumor growth with respect to tumor size in our study could be explained by the enhanced function of NK cell cytotoxicity to tumor cells despite the lower NK cell signals after combined cordycepin and anti-PD1 therapy. Taken together, the in vivo activity of cordycepin in augmenting anti-PD1 efficacy may be mediated by various effects, including anti-cancer [32], immunomodulatory [11], and suppressive effects on NK-A2AR and NK-A2BR expression. Therefore, further large-scale studies are necessary to determine the mechanisms underlying the effects of the cordycepin and anti-PD1 therapy.

Here, the expression of macrophage-A2AR in the spleen was induced after anti-PD1 treatment alone but not after combination therapy with cordycepin and anti-PD1. This suppressive impact on macrophage-A2AR expression in the spleen after combined cordycepin and anti-PD1 therapy may have enhanced the A2AR blockade after cordycepin treatment. 

The percentage of A2AR-expressing macrophages among CD3+/CD45+ cells was lower in the TME than in the spleen, and macrophage A2AR expression in the tumor was significantly higher after combined therapy with cordycepin and anti-PD1 than after anti-PD1 treatment alone. However, the proportions of M0, M1, and M2 macrophages among the tumor-associated macrophages in the TME could not be identified in this study. Therefore, confounding factors, such as M2 macrophage induction [33], may have contributed to the increased macrophage A2AR expression in this study, warranting further investigation.

The structural difference between adenosine and cordycepin, a derivative of adenosine, is that cordycepin lacks the hydroxyl group in the 3′ position of its ribose part. Although some biological similarities were found between these two compounds, many more differences in terms of anti-cancer effects and immunomodulation were evident, suggesting that this functional group might be important for augmenting the anti-PD-1 effect and might be tested as a target for further chemical modification. (Figure S1).

## 5. Conclusions

In conclusion, this study revealed not only the direct inhibitory effect of cordycepin on cell growth but also its novel role in enhancing the immune response against mouse colorectal carcinoma CT26 cells. The suppressive effects of combination therapy with cordycepin and anti-PD1 on CT26 cells in vivo were significantly greater than those of cordycepin or anti-PD1 treatments alone. Moreover, the body weight, liver function, renal function, and WBC count of the mice demonstrated the safety and efficacy of this combination treatment, indicating that this combination therapy against CT26 cells was applicable and well tolerated. Notably, cordycepin augmented anti-PD1 efficacy in vivo via a suppressive effect on NK-A2AR and NK-A2BR expression and enhanced the inhibitory effects of anti-PD1 on A2AR and A2BR expression in splenic macrophages and intratumoral NK cells, indicating a novel pathway to clarify the biological function of cordycepin in colon cancer.

## Data Availability

Data are contained within the article.

## Supplementary Materials

The following supporting information can be downloaded at https://www.mdpi.com/article/10.3390/biomedicines12071568/s1. Figure S1. Chemical structure of adenosine (A) and cordycepin (B). Cordycepin, also named 3’-deoxyadenosine, is a derivative of adenosine, differing from the latter by the lack of the hydroxy group in the 3’ position of its ribose part [34].
