# Supplementary material for: Cordycepin Augments the Efficacy of Anti-PD1 against Colon Cancer
Source: Biomedicines. 2024 Jul 15;12(7):1568. doi: 10.3390/biomedicines12071568 (PMC11274779; doi:10.3390/biomedicines12071568)
Supplement: Supplementary file 1 [file biomedicines-12-01568-s001.zip › biomedicines-3047810-supplementary.pdf]

Supplementary Material

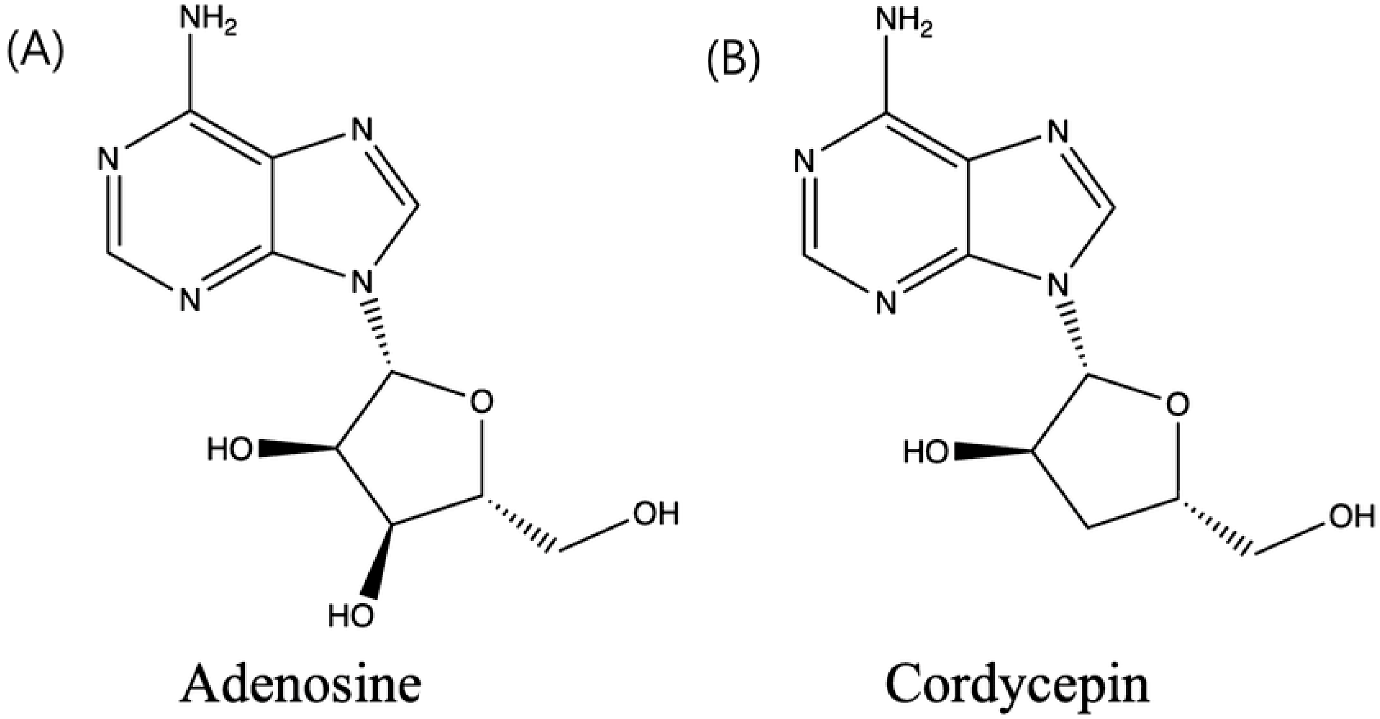

Figure S1. Chemical structure of adenosine (A) and cordycepin (B). Cordycepin, also named 3'-deoxyadenosine, is a derivative of adenosine, differing from the latter by the lack of the hydroxy group in the 3′ position of its ribose part [34].
